# Dental Work among the Poor: How Can It Best Be Accomplished?

**Published:** 1900-09

**Authors:** H. A. Kelley

**Affiliations:** Portland, Me.


### DENTAL WORK AMONG THE POOR: HOW CAN IT BEST BE ACCOMPLISHED?[1]

BY H. A. KELLEY, D.M.D., PORTLAND, ME.

I AM pleased to appear before you to discuss the question of dental work among the poor, because it is a question that, however much you may individually have thought upon it, if I am to judge by my reading of the dental journals, has received almost no attention at our society meetings.

I hope to make this a discussion in which this paper will be only the opening, for my study of the subject has not left me with well-defined opinions which I can give you with a reasonable hope that they are right and final, nor has the practical experience I have had in this work given results any more emphatic. In my

---

[1] Read before the Northeastern Dental Association, October, 1899.

consideration of the social and ethical side of the question those of you who are familiar with your Spencer, Huxley, and Mills will recognize that I have drawn from their works quite largely. Also I shall be obliged to consider general charity and its data, and from that reason to dental charity, because there are no data upon dental charity at my hand except the small amount deducted from my own experiments.

I have said this subject has received but little attention at our society meetings, but in 1896 R. C. Newton, M.D., read a paper, which stands out almost alone, before the New Jersey State Dental Society, entitled "What shall be done for the Teeth of the Poor?" Dr. Newton says, "A poor man can have his every bodily ailment and deformity gratuitously treated, and that, too, by the best available skill, except his teeth,—perhaps the most generally important and necessary of all the special organs. This long-felt want will surely be filled. . . . The higher position a man attains in the medical profession the more time and thought must be given to charitable work. So the dentists, if they wish to be esteemed by the public generally as specialists of medicine, must give their time and skill to treating the poor. They must do as the oculists and the aurists do or give up, in some measure at least, the place which they deserve to hold among their professional brethren. It is willingness to give time, thought, and skill to the service of the poor which has elevated and ennobled the profession of medicine. It is this that has made it the most generally beloved and respected of all the professions."

In July, 1896, I read a paper before the Maine Dental Society entitled "What Dentistry owes the People." In that paper I said, "When a profession is known, or believed, to be working for humanity and not for hire alone, then it is upon a high plane not only in its own estimation, but in the estimation of the people of the world. Because you need my services I give them to you, not because you pay me money. This is the spirit in which we should work. Now, dentistry does owe the people something, and when we give them that something we will stand beside the liberal professions. You all know that dentistry is doing almost nothing for the poor. We have no charitable hospitals. You and I do something in the charitable line, but are we doing all that we owe the poor? I think not."

I will not quote further from that paper now, but I introduced it here for the double purpose of showing you, first, that at the same time Dr. Newton was reading his paper I was writing mine, and that his thoughts were nearly repeated by one of the profession. As a result of my work, of which this paper was a part, a free dental clinic was opened in Portland, Me. Let me quickly sketch my experience in this work, that you may know something of the preparation I have had before writing this paper. After it occurred to me that dentistry owed the people something I started to help pay the debt.

After some preliminary work the paper I have mentioned was written and read before the Maine Dental Society, and afterwards a popular edition, if I may so term it, was published in one of the Portland daily papers as an aid to securing subscriptions for a dental infirmary.

Enough money having been subscribed, and a home having been offered us at the Maine Eye and Ear Infirmary, a free dental clinic was established. This infirmary had free clinics, in the out-patient department, in all the specialties of medicine except dentistry, and it was thought best to add our dental clinic to this institution. With the money donated we purchased our special instruments and material. Drs. D. W. Fellows, S. A. Packard, and myself were elected Surgeons in Oral Surgery and Dentistry in the out-patient department of the Maine Eye and Ear Infirmary, and we began our operations, each giving two clinics a week. After a year of this work I again read a paper before the Maine Dental Society entitled "A Year's Work among the Poor," in which I gave an account of the workings of our clinic. And now, gentlemen, I come before you with the experience and preparation to discuss Dental Work among the Poor; How can it best be accomplished?

Marcus Aurelius Antoninus quotes Democritus as saying, "If you would live at your ease, manage but a few things," and then says, "I think it had been better if he had said, 'Do nothing but what is necessary and what is in accord with the reason of a social being.'" For by this rule a man has the pleasure of making his actions good. For the greater part of what we say and do being unnecessary, if this were but once retrenched we should have both more leisure and less disturbance. And therefore before a man sets forward he should ask himself this question, "Am I not upon the verge of something unnecessary?" I asked myself this question three years ago, as I may show, and also my reply, by the following from my paper: "I simply mention these things (some of the evils of charity) to show I realize the dangers of charity as practised to-day. But however far charity may have been overdone in this world, surely dentistry has not transgressed that way." That was my answer three years ago. I shall answer it again to-day in the light of my added experience.

That you may not think I have suddenly changed my views, let me quote from my second paper reporting my year's work at the clinic: "It was when we came to actually begin operations, however, that we found ourselves face to face with many serious questions, and some of these questions I have not been able to answer to my satisfaction yet." Nor has the past year answered them, though I feel somewhat more sure that my beliefs are right. Lecky says charity really came into the world with Christianity, and it is interesting to note that as soon as it arrived came the abuse of it. "Long before the era of Constantine (A.D. 274-337) it was observed that the charities of the Christians were so extensive —it may perhaps be said so excessive—that they drew very many impostors to the church." And it is interesting, while we are quoting Lecky, to read that it was a Roman lady named Fabiola who in the fourth century founded in Rome, as an act of penance, the first public hospital; as it tells us, medicine began its charitable work early in the history of charity.

That there is need of charitable work in all the walks of life there can be no question, but is this charitable work being done in the way that would best advance society? In early societies there went, along with the dependence of inferiors, a certain kind of responsibility for their welfare. Along with gradual substitution of the system of contract for the system of *status* this relation has been changed in such a manner that while the benefits of independence have been gained, the benefits of dependence have been lost. The poorer citizen has no longer any one to control him, but he no longer has any one to provide for him. So much service for so much pay, and the money having been paid for the service, no further claim is recognized. The ancient *régime* of protection and fealty has ceased, while the modern *régime* of beneficence and gratitude has but partially replaced it. May we not infer with tolerable certainty that there has to be reinstated something akin to the old order in a new form? Already such moral ties are in some measure recognized. Already all householders moderately endowed with sympathy feel bound to care for their servants during sickness, and even corporations, that are said to have no souls, make provisions for their help, *vide* the new rules of the Pennsylvania Railroad.

The sole requisite seems to be that usage which thus shows itself here and there irregularly should be called into general activity by the gradual disappearance of artificial agencies for distributing aid.

Besides re-establishing these closer relationships between superior and inferior which during our transition from ancient slavery to modern freedom have lapsed, and besides bringing beneficence back to its normal form of direct relation between benefactor and beneficiary, this personal administration of relief would be guided by immediate knowledge of the recipients, and the relief would be adjusted in kind and amount to their needs and their deserts.

When the responsibility fell directly on each of those having some spare means, each would see the necessity for inquiry and criticism and supervision; so increasing the aid given to the worthy and restricting that given to the unworthy. People who think that the relations between expenditure and production are so simple naturally assume simplicity in other relations among social phenomena. Is there distress somewhere? They suppose nothing more is required than to subscribe money for relieving it. On the one hand, they never trace the reactive effects which charitable donations work on bank accounts, on the surplus capital bankers have to lend, on the productive activity which the capital now abstracted would have set up, on the number of laborers who would have received wages and who now go without wages; they do not perceive that certain necessaries of life have been withheld from one man, who would have exchanged useful work for them, and given to another, who persistently evades working. Nor, on the other hand, do they look beyond the immediate mitigation of misery. They deliberately shut their eyes to the fact that as fast as they increase the provision for those who live without labor, so fast do they increase the number of those who live without labor; and that with ever-increasing distribution of alms there comes an ever-increasing outcry for more alms. And we must remember the first charity a person will accept is free medical treatment. Persons that cannot be induced to ask for free food or fuel or clothes will with avidity embrace the opportunity to obtain free medical treatment, and the acceptance of free medical attendance is the first step towards pauperism.

There is already a tendency towards what is generally known as nationalism,—a belief among the common people that the city and the State owe them a living, and that medical attendance, among other things, should be furnished them by common taxation, regardless of their financial standing as individuals. I will not use much time on this phase of the question, for I do not think it worthy of it here, but let me give you just one item. One investigator of the situation in the West, about the time of the last presidential election, gave it as his honest opinion that uncharitable charity is more responsible for the condition of things there than the silver question or the tariff question or anything else. And he said further that "the people of the West (the common people at least) look to Washington and its legislation to remedy the condition in the West when help can only come from within themselves."

Less objectionable than administration of poor relief by a law established and coercive organization is its administration by privately established and voluntary organizations, says Spencer. For, though the vitiating influences of coercion are now avoided, the vitiating influences of proxy distribution remain.

The beneficiary is not brought in direct relation with the benefactor, but in relation with an agent appointed by a number of benefactors.

The transaction, instead of being one which advantageously cultivates the moral nature on both sides, excludes culture of the moral nature, as much as is practicable, and introduces a number of bad motives.

With this study of general charity let us approach the study of medical charity work, our nearest type, and dental charity work, our special field.

Medical charity work is growing out of the "individual ministration" that Spencer calls "the normal form of ministration" into beneficence administered by compulsory, or more often non-compulsory, social machinery. And I venture to say it was this individual ministration of the older practitioners that made medicine the most generally beloved and respected of all the professions, and not the machinery of the hospitals and the infirmaries.

What I know of medical hospitals and infirmaries makes me doubt if this tendency of the medical profession is in the right direction. But this is perhaps the one question more than any other I would not like to try to answer definitely to-day.

That hospitals and infirmaries are doing great good there can be no doubt, nor, again; can there be any doubt they are doing immense harm. Spencer, after considering the evils of general charity, says, "Nor is it otherwise with institutions thought by most people to be indisputably beneficial,—hospitals and dispensaries."

The first significant fact is that thirty per cent. of the people of London are frequenters of them, and the largeness of this proportion makes it clear that most of them are able to pay their doctors.

*Gratis* medical aid tends to pauperize in more direct ways. The out-patient begins by getting physic, and presently they get food; and the system leads them afterwards to openly solicit pecuniary aid. This vitiating effect is proved by the fact that during the forty years of 1830 to 1869 the increase in the number of hospital patients in London has been five times greater than the increase of population, and as there has been but a very small, if any, increase in disease, the implication is obvious. Moreover, the promise of advice, by the best available skill, for nothing attracts the mean-spirited to the extent that the poor are now being gradually ousted out of the consulting-room by well-to-do persons,—twenty per cent of the out-patients in one hospital having given false addresses for the purpose of concealing their identity. Most of these were without doubt impostors, though pride may account for a few.

Two papers written the last year by B. S. Coler, the Comptroller of New York City, and F. H. Giddings, Professor of Sociology in Columbia University, give me the means of studying the charity work of that city. Giddings says, "The appropriation of public money to private institutions has become a scandalous abuse, but we shall never understand its strength until we frankly face the fact that the public has been experimenting with it, hoping thus to find a way of escape from the greater abuse that attends the administration of public relief by public agencies, except when they are incessantly watched and held up to the broadest light of publicity by the disinterested efforts of private citizens. If private organizations are encouraged to do all in their own power under a system wherein the State grants them aid under strict conditions, lays down necessary rules for their government and guidance, and remorselessly exposes all their transactions, the actual result may be better in the long run than if State and private associations proceed independently of one another, often duplicating each other's work, or, if not that, working at cross purposes."

Coler says, "It is easier for an industrious and shrewd professional beggar to live in luxury in New York than to exist in any other city in the world." New York City gives annually five million dollars directly and two million dollars indirectly to public charity. This is almost twelve per cent. of the money raised by taxation for city purposes proper.

More than three million dollars of this money is paid to private institutions and societies over which the city has no control. As the city pays one hundred and ten dollars per year for the support of a child and a hundred and fifty dollars per year for an adult, and as the children are in excess, this sum would feed and clothe more than forty thousand people. Of the five million that New York City spends directly in charity, two million is absorbed by salaries and expenses, some institutions spending sixty to eighty per cent. of the money granted in salaries and one ninety-four per cent. These figures show something of what Spencer calls the cost of social machinery; and, Coler says, there is no evidence they are dishonest, but they are, as a rule, conducted by men and women whose motives are good, but who have no experience or practical knowledge to fit them for the management of a charitable institution. They are easily imposed upon by professional beggars, and in most cases fail in their well-meant efforts to reach and relieve the deserving who are in actual need.

Coler admits that when the city expends its money itself it does not do much better, for of the two million spent that way five hundred thousand is paid for salaries. For every five dollars paid by the city treasurer to relieve the sick and destitute two dollars is absorbed by the salary and expense account. As seen by Coler, the chief abuses of the present system of public charity "are the extravagant expenditures for salaries and the steady and rapid increase of pauperism, due to the misdirected efforts of the inexperienced persons who control so many of the smaller societies that receive city money." How great this increase has been is shown by the fact that of the two hundred and twenty charitable societies of New York helped by the city over one hundred have been organized in the past ten years.

Many of the great American cities can show a better result than New York, but Giddings says this is due almost wholly to the enormous extension of private as over against public charity in these other cities.

These experiences unite to show that whatever benefits flow from hospitals and infirmaries are accompanied by grave evils,—evils sometimes greater than the benefits.

They force on us the truth that, be it compulsory or non-compulsory, social machinery wastes power and works other effects than those intended. In proportion as beneficence operates indirectly instead of directly, it fails in its end, says Spencer. Beneficence which takes the form of giving material aid to those in distress has the best effects when individually exercised. It is true, however, individual beneficence often falls short of the requirements, often runs into excesses, and is often wrongly directed. At any rate, it must be admitted that individual ministration to the poor is the normal form of ministration; and that made more thoughtful and careful, as it would be if the entire responsibility of caring for the poor devolved upon it, it would go a long way towards meeting the needs, especially as the needs would be greatly diminished when there had been excluded the artificially generated poverty with which we are surrounded.

"Within the intricate plexus of social relations surrounding each citizen there is a special plexus more familiar to him than any other. Every one who can afford to give assistance is brought by his daily activities into immediate contact with a cluster of those who are severally liable to fall into a state calling for aid; and there should be recognized a claim possessed by each member of this particular cluster."

Can the evils be eliminated, and would hospitals and infirmaries then be the best media for extending medical and dental aid to the poor? I have my doubts whether all the evils can be eliminated, but I am quite certain if they could we would have in them the best system for doing charitable work in medicine and dentistry as well.

Where my doubt arises is, with the evils it seems possible to eliminate removed and those it seems impossible to eliminate remaining, would hospitals and infirmaries then be the best solution of the question? Of course the greatest evil is the use of them by the people who are able to pay, thus working all the harm that any misdirected charity does. How great this use of them by the well-to-do people is I have shown. I believe this evil can be remedied. Much thought is now being given to this subject, and it seems simple enough to devise a method that will make at least a vast improvement here.

Dr. Clarence J. Blake, of Boston, in a paper entitled "A Hospital Clearing House," read before the Massachusetts Medical Society last June, recommends a clearing house for hospitals, and says, "A clearing house in other lines of business (than banking) has come to be a central bureau not only for the establishment and maintenance of balances, but for such co-operative investigation as may constitute it a bureau of information as well; and it is readily conceivable that the establishment and maintenance by hospitals in a large city of an officer to whom, or a bureau to which, there could be referred, for more leisurely investigation, pecuniarily doubtful cases, would not only relieve the individual administrator of a part of a most unwelcome task, but would in time become a channel through which there should flow interchange of ideas, and from which there should emanate to the public instructive information as to the rights as well as the obligations of hospitals."

Dr. Emma Culbertson, some years ago, referring to the exclusion of the unworthy from these charitable institutions, says, "The sifting out of improper cases is a complicated and delicate task. Experience seems to prove that it is better to delegate such investigation to a single official in each institution. Much economy of effort, however, with greater increased efficiency, will result from the establishment of a central bureau to which all charitable institutions of the city should report, and in which the information collected will be easily accessible." This would make it impossible for impostors refused at one institution to obtain admission at another less careful, and establish a black-list of impostors.

Another very grave evil is, the clinicians are responsible for the admission of large numbers of impostors through their unseemly desire to show in the annual report large clinics instead of aiming at pure ones. They should be encouraged in their efforts to do a large amount of charitable work, but nothing can excuse them for taking as charity patients those known to be able to pay, or, if there is only a suspicion, of taking them without thorough investigation.

Still another evil is that so many of the hospitals and infirmaries either receive a patient absolutely without charge or else will not receive him at all. True charity demands that each case should be individually examined, and that the patient should pay as near the regular charge as he is able. Where this money should go I have not the time to discuss. I believe that many institutions are now charging a small fee, but this is a very serious question.

Then there is the vast amount of money spent in the hospitals which represents a part of what Spencer calls "the cost of social machinery." Could the money it costs to build and maintain our hospitals and infirmaries be better used if spent directly upon the poor? Spencer thinks it could, but I believe it very doubtful. I think the first three evils I have mentioned are the greatest evils of the hospitals, and with these eliminated I think we would have the model which dentistry should use in its charitable work.

Now to return to our clinic in Portland. As I have said, there was no attempt to advertise the fact we had one. We believed all the work we could do would find us. We informed the charitable institutions of our city of its existence, and in this way secured patients of known need from the St. Elizabeth Orphan Asylum, the Maine School for the Deaf, the Temporary Home, and the Gospel Mission, all charitable institutions of known worth. Also the contributors to our fund were requested to send to us any persons known by them to be needy, and workers in charity throughout our city were asked to remember we were there and always glad to be of use to them and their wards. In this way our clinic was remarkably free from impostors, and I wish it could have continued, but as the Eye and Ear Infirmary desired our room we are now without a home. We are not able to maintain rooms for ourselves, and so perhaps this experiment is at an end. However I may feel in regard to other hospitals and infirmaries, I know this one was a remarkably pure clinic, and that a very small percentage of patients received were impostors. But then it was a very small clinic, and one that probably would not so early attract the attention of impostors. Possibly—most likely, if it had been larger and wider reaching it would have had all the evils of the other clinics.

I wish I had time to tell you more about this clinic, but it is not my purpose to make that a part of this paper. Did I feel strongly that such an infirmary worked only for good, I would try to re-establish this dental clinic. With the doubts I hold and have here expressed, I hesitate. But Huxley says, "Society is stable when the wants of its members obtain as much satisfaction as, life being what it is, common sense and experience show may be reasonably expected," and I am not sure dental society in Portland is stable with this dental clinic out of existence.

If we do not consider dental infirmaries as the best means of doing our charity work, then it only remains for us individually to do that which we can to alleviate the sufferings of the poor. And I really think we can more carefully regulate our charity work, each at his own office, than it is done for us as a part of any hospital or infirmary as now managed. Again, we can without question at our office fix a fee, regulated by the necessity of the sufferer, and that is a very difficult thing to do at charitable institutions. Possibly we will be altogether much less imposed upon at our offices than at the infirmaries; but, on the other hand, we lose much if we lose the infirmaries, for it would be difficult to set apart certain definite hours in the week when you would receive charity patients. It would be annoying to have them coming in at any time, and you would not like to have the average charity patient in your reception-room together with your regular patients. I warn you charity patients are just as sensitive to slight as your most aristocratic patient.

I am not sure but that we should give them the same attention as our best patients receive, for it is a great question as to how far the fact of their being charity patients should be shown to them. We should not wish to take away their self-respect, and yet we ought to impress upon them some way that they are in a condition they must strive to rise above, a position in which it is not pleasant to remain.

I am afraid my paper is already too long, and I only hope I have suggested to you some of the benefits and disadvantages of the methods of doing dental charity work, and that you will all help to do something for the poor; for, believe me, there is a great need that they be taught how to care for their teeth and assisted in doing it by competent dentists.

Let me close with a quotation, that I may in my ending again suggest to you that this paper is intended more as opening a discussion than as presenting ideas of mine known to be truths. Spencer says, "Hence it is not to be expected that modes of thinking on social affairs are to be in any considerable degree changed by whatever may be said respecting the social science and its difficulties. The only hope is that here and there one may be led, in calmer moments, to remember how largely his beliefs about public matters have been made for him by circumstances, and how probable it is that they are either untrue or but partially true.

"When he reflects on the doubtfulness of the evidence he generalizes; when he counts up the perverting sentiments fostered in him by education, country, class, party, creed, when observing those around, he sees that from other evidence selected to gratify sentiments partially unlike his own there result unlike views; he may occasionally recollect how largely mere accidents have determined his connections.

"Recollecting this, he may be induced to hold these convictions not quite so strongly; may see the need of criticism of them with a view to revision, and, above all, may be somewhat less eager to act in pursuance of them."

